# Analysis of 1.2 million foot scans from North America, Europe and Asia

**DOI:** 10.1038/s41598-019-55432-z

**Published:** 2019-12-16

**Authors:** Ales Jurca, Jure Žabkar, Sašo Džeroski

**Affiliations:** 1 Volumental AB, Stockholm, Sweden; 2 Jozef Stefan International Postgraduate School, Ljubljana, Slovenia; 3 Faculty of Computer and Information Science, University of Ljubljana, Ljubljana, Slovenia; 4 Jozef Stefan Institute, Ljubljana, Slovenia

**Keywords:** Anatomy, Musculoskeletal system

## Abstract

For decades, footwear brands have developed products using outdated methods and measurements, working with limited insight into the foot shapes and dimensions of their target customers. The integration of 3D scanning technology into footwear retail stores has made it possible for this research to analyze a database containing a large number of male and female 3D foot scans collected across North America, Europe, and Asia. Foot scans were classified into length classes with 5mm length increments; mean width, instep height, and heel width were calculated for each length class. This study confirms the existence of many statistically significant differences in mean foot measurements amongst the regions and between the sexes, and a large dispersion of foot measurements within each group of customers. Therefore, shoes should be developed separately for each group, region, and sex, and at least 3 shoe widths per length class are required to provide a proper fit for 90% of customers. Beyond this, our analysis asserts that a shoe designed for a single group will fit a different segment of the population in another group, and that existing last grading tables should be updated to reflect the foot dimensions of current consumers.

## Introduction

Proper fit is an essential customer expectation of footwear. In order to provide a good fit to different foot shapes and dimensions, shoes are available in multiple length sizes, with some available in multiple widths per length size. The mondopoint, European, UK, and US sizing systems are most commonly used to indicate the size of a shoe. A full size length increment is 5 mm in the mondopoint system and 6.67 mm in the European sizing system. Most shoes in the UK and US sizing systems are available in half sizes with a half size length increment of 4.23 mm. The shoe last is the primary determinant of the inner shape of a shoe. This is a wooden, plastic, or metal object with a shape similar to that of a human foot, used to form a shoe during the shoe manufacturing process. Shoes and shoe lasts are commonly developed in one size and width, called “sample size”. The complete size range of a shoe is developed by grading - the process of scaling the sample size shoe and the shoe last to develop other sizes and widths^1^. Scaling factors are defined with grading tables. Length increments between two consecutive sizes are well defined for each sizing system^2^. Width increments between two consecutive sizes are most commonly constant across the whole length size range^1^. Furthermore, width increments between two consecutive widths of the same size are constant for the whole size and width range. Grading tables haven’t changed for decades and it’s not clear what data they are based on.

Information about foot dimensions and shapes should be used in the footwear development process in order to produce shoes that fit the target customers. Previous studies have analyzed foot shapes and measurements, and some studies have identified sex-related differences in foot dimensions. In the USA, the feet of the male and female subjects differ significantly in 11 distinct foot measurements^3^. The feet of female subjects are relatively higher, but narrower than those of male subjects in the USA^4^. In Europe, Australia, China, and Taiwan, the feet of female subjects are lower and narrower compared to the male feet of similar foot length^5–8^. Other studies have identified differences between geographical regions. The shape of the forefoot of male subjects from Korea and Japan differs from that of North American male subjects^9^. The feet of the Japanese male and female subjects are wider than the feet of the Australoid and Caucasoid subjects of similar foot length^10^. Female Japanese subjects have significantly different forefoot shape than Taiwanese subjects of similar foot length^11^. The methods of measuring feet in previous studies differ in the measurement tools and weight-bearing conditions used. Some studies have used manual or digital calipers and measuring tapes, others have used 3D foot scanners from several different vendors; therefore, results are difficult to compare between most previous studies^12^. Manual foot measurement methods have lower precision and accuracy than 3D scanning technology^12^. In most of the previous studies, body weight was equally distributed on both feet while feet were measured; in some studies, feet were measured in full weight-bearing condition. Methods of analyzing foot measurements also differ in previous studies. Some researchers have divided foot measurements per length class and compared feet within each length class, while some have compared all feet regardless of foot length, and others still have normalized feet before comparing them. Normalizing foot measurements by foot length and analyzing relative foot measurements is not recommended since information essential for grading tables is lost in the normalization step^13^. All the differences in foot measurement and analysis methods between previous studies make it very difficult to compare results between the studies.

Previous studies have analyzed small data sets of foot measurements, limited to smaller geographical regions. An overview of previous foot measurement studies, including a bibliographic reference, the year of the publication, the number of feet that were measured in each study, sex, nationality, race of subjects included in each study, and measurement methods, as well as the measurements of the present study that were included in the previous studies are presented in Table 1. The studies are listed in chronological order, starting from the earliest study at the top and with the present study at the bottom.

**Table 1:** An overview of previous foot measurement studies, characterized in terms of the year of study publication, number of measured feet, sex, location of subjects, and measurement methods, as well as the measurements of the present study that were included in the previous studies (L: length, W: width, I: instep height, H: heel width).

Study	Publ.	Number of feet	sex of subjects	Countries/races	Measurement method	Measurements
Hawes *et al*.^9^	1994	1,221	male	North America, Japan and Korea	a digital caliper	L,W
Hawes *et al*.^17^	1994	1,197	male	Caucasian	a sliding caliper	L,W,I,H
Kouchi^10^	1998	3,208	male and female male and female	Japan, Indonesia, France, Australia	a scriber and a measure tape	L,W,H
Mochimaru *et al*.^18^	2000	56	female	Japan	plaster models and a 3D digitalizer	L,W,I,H
Wunderlich *et al*.^3^	2001	1,568	male and female	USA	a caliper and a measure tape	L,W,I,H
Xiong *et al*.^19^	2008	50	male and female	Hong Kong	Vorum 3D scanner	L,W,I
Krauss *et al*.^5^	2008	1,590	male and female	Europe	Pedus 3D scanner	L,W,I,H
Gangming *et al*.^4^	2009	90	male and female	USA	plastec casts and an optical scanner	L,W,I,H
Krauss *et al*.^20^	2010	910	female	Europe	Pedus 3D scanner	L,W,I,H
Mickle *et al*.^6^	2010	624	male and female	Australia	Infoot 3D scanner	L,W,I,H
Jurca *et al*.^16^	2010	9,220	male and female	Europe	Infoot 3D	L,W,I,H
Hong *et al*.^7^	2011	2,321	male and female	China	Ariel Motion analysis system	L,W,I,H
Krauss *et al*.^13^	2011	574	male and female	Caucasian	Pedus 3D scanner	L,W,I,H
Rodrigo *et al*.^21^	2012	50	male and female	Hong Kong	Vorum 3D scanner	
Domjanic *et al*.^22^	2013	166	female	Croatia	Pedus 3D scanner	
Lee *et al*.^12^	2014	130	male and female	Taiwan	digital caliper, Infoot 3D scanner, digital footprint and ink footprint	L,W,H
Lee *et al*.^11^	2015	42	female	Taiwan and Japan	Infoot 3D scanner	L,W,I,H
Lee *et al*.^8^	2015	3,000	male and female	Taiwan	Infoot 3D scanner	L,W,I,H
Baek *et al*.^23^	2016	350	N/A	South Korea	NEXCAN 3D scanner	L,W,I,H
Stankovic *et al*.^24^	2018	124	male and female	Belgium	FootIn3D scanner	
Wannop *et al*. ^25^	2018	2,902	male	North America	several 3D scanners	L,W,H
**The present** **study**	**2019**	**1,200,847**	**male and female**	**North America, Europe and Asia**	**Volumental** **3D scanner**	L,W,I,H

The recent installation of 3D foot scanning technology into footwear retail makes it possible to collect large data sets of 3D scans of customers’ feet from various geographical locations. These data sets provide new opportunities for research of foot dimensions and shapes. The aim of this study was to perform an analysis of foot lengths, widths, instep heights, and heel widths extracted from 3D foot scans, and provide new insights to footwear developers so that the products will have a higher likelihood of fitting the target customers’ feet. The study was performed on a large data set of 3D foot scans acquired in North America, Europe, and Asia. Differences in analyzed foot dimensions between the 3 regions were studied separately for male and female feet in order to identify differences between the regions. Male and female feet were compared within each region to identify sex differences in foot measurements.

## Methods

### Subjects

This study was conducted in compliance with the Helsinki Declaration, and has been approved by the Swedish Ethics Review Authority (reference No. 2019-03243). The study was conducted on foot scans collected by Volumental AB prior to the study. The subjects in the study were customers of 712 brick and mortar stores of footwear brands and retailers that are using a Volumental foot scanning solution. Customers came to the stores to buy shoes; 3D foot scanning was a standard step of the shoe buying process in these stores. Foot scans, collected between November 1^*st*^, 2017 and August 31^*st*^, 2018 from the following global regions were used in the study: North America (Canada and USA), Europe (Andorra, Austria, Czech Republic, Denmark, Finland, France, Germany, Hungary, Italy, Latvia, Lithuania, Netherlands, Norway, Poland, Romania, Russia, Slovakia, Slovenia, Spain, Sweden, Switzerland, and the United Kingdom) and Asia (China, Japan, Korea, Malaysia, Singapore, Taiwan, and Thailand). Before scanning their feet, the customers were given a chance to read a Volumental privacy policy, where it was stated that their body measurement data could also be used for research purposes. Since the foot scans were collected in a retail environment, the process had to be quick, simple, and user-friendly; therefore, the only attribute that was stored for every customer was sex.

### Measurement procedure

Volumental 3D foot scanners were used to scan both feet of each customer. A sales associate uses a tablet to interact with the scanner during the foot scanning process and to show the scan results to the customer. A customer needs to take off their shoes before scanning and pull up their pant legs. They can leave their socks on or go barefoot. Socks of any color or material can be worn by the customer during scanning. The customer stands on the base platform of the scanner with both feet positioned slightly apart from one another. Body weight should be equally distributed between both feet. The sales associate selects sex and starts a scan. To capture both feet, the scanning process takes 5 seconds. After the scan is successfully processed, 3D meshes and foot measurements of both feet are displayed to the customer and used for recommending well-fitting footwear. The software algorithm detects whether the customer was scanned barefoot or wearing socks. In order to avoid any influence of various socks thicknesses on feet measurements, only barefoot scans were used in the study. All scanners were connected to the internet, which enabled automatic transfer of 3D mesh files to a cloud server where the foot mesh files were anonymized and stored for further analysis.

### Foot measurements

Precise definitions of foot measurements are crucial for achieving repeatable and reproducible results, regardless of the measurement technology being used to scan feet^14,15^. A detailed explanation of the measurement extraction algorithm enables comparisons of results of future studies with the results of this study. It is important to use a consistent foot orientation to get repeatable foot measurements, regardless of the foot orientation on the scanner. Foot orientation and measurement extraction algorithms used in the European project Dorothy^16^ were optimized for automatic extraction of foot measurements without the need for placing anatomical landmarks on feet prior to scanning. The algorithms were developed empirically based on a large number of tests^16^, with the objective of extracting the most relevant foot measurements that were later used by footwear brands. The methods of the Dorothy project have been documented thoroughly. The Volumental scanners extract foot measurements from 3D meshes using the methods as were used in the Dorothy project; therefore, the same methods were used in this study.

**Figure 1:**
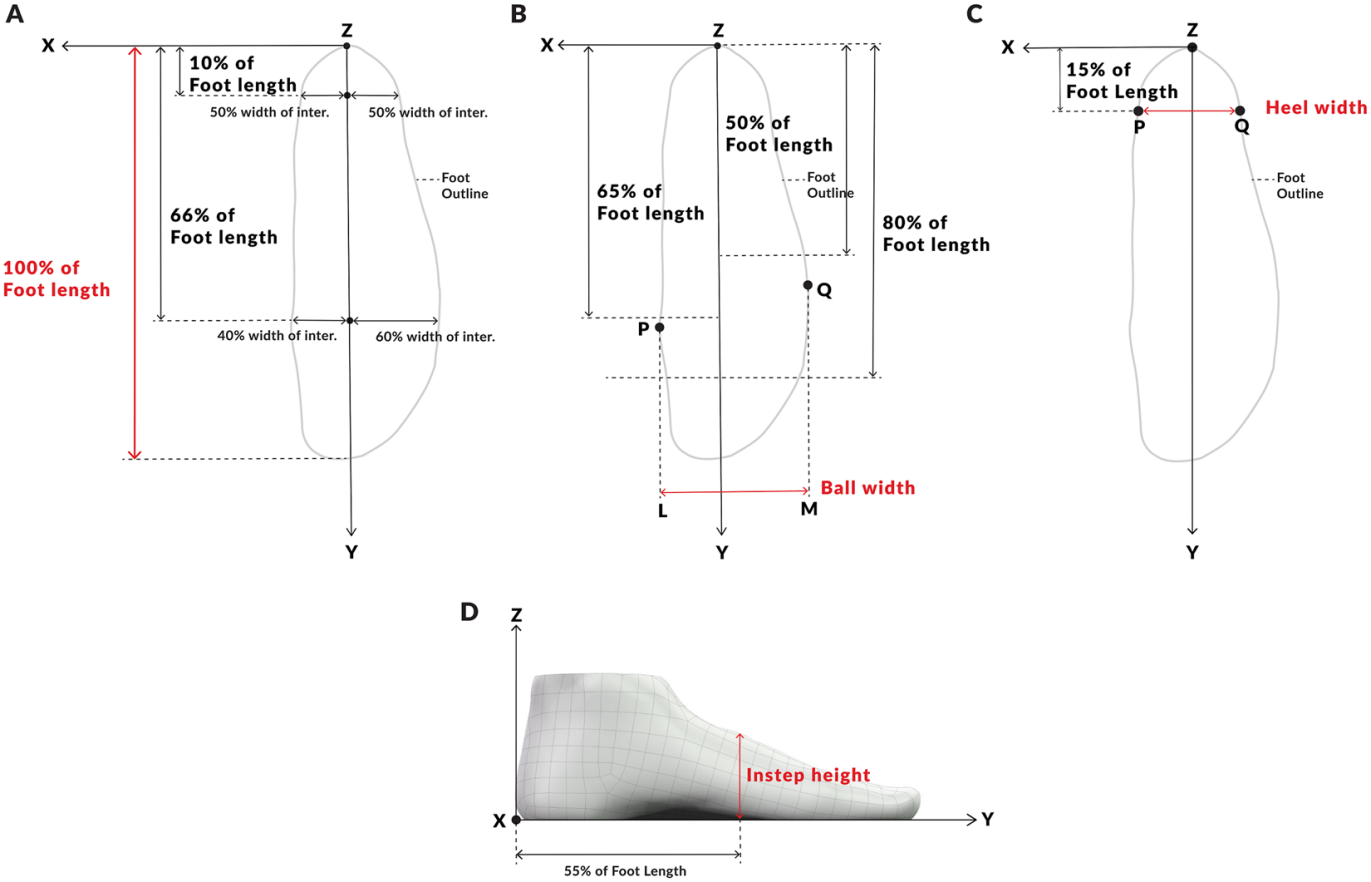
Foot dimensions definitions. (**A**) Foot orientation and foot length. (**B**) Foot width. (**C**) Heel width. (**D**) Instep height.

The X-Y plane of the foot coordinate system was aligned with the base platform of the scanner; therefore, points on the plantar part of feet were touching the X-Y plane. Foot scans were oriented using the foot outline (vertical projection of the foot to the X-Y plane) as presented in Fig. 1A. In order to exclude ankle bones from the foot outline, only points of the foot mesh that are closer than 30 mm to the X-Y plane were used in the vertical projection. The main foot axis Y was defined by two foot outline cross sections that were perpendicular to the main foot axis. A cross section at 10% of the foot length (in the heel area) was divided by the foot axis to 50% on the medial side and 50% on the lateral side. A cross section at 66% of the foot length (in the forefoot area) was divided by the foot axis to 40% on the medial side and 60% on the lateral side. The following foot dimensions were extracted from each 3D mesh: foot length, foot width, instep height, and heel width. Foot length was defined as the length of the foot outline bounding box along the main foot axis.

Foot width was defined using points P and Q as presented in Fig. 1B. Point P was the utmost point of the medial part of the foot outline, in the region between 65% and 80% of the foot length. Point Q is the utmost point of the lateral part of foot outline, in the region between 50% and 80% of the foot length. Foot width was defined as the distance between the lines that were parallel to the main foot axis and were passing through points P and Q. Heel width was defined using a foot outline cross section that was perpendicular to the main foot axis, located at 15% of the foot length, as presented in Fig. 1C. Heel width was equal to the distance between the two points on the cross section.

Instep height was defined using a foot mesh cross section that was perpendicular to the main foot axis, located at 55% of the foot length, as presented in Fig. 1D. Instep height was equal to the largest distance of any point on the cross section from the X-Y plane.

### Data analysis

Foot scans were grouped by the three geographical regions and by sex within each region. Furthermore, scans of each group were classified into length classes according to the mondopoint sizing system^2^. Length classes indicate the foot length in mm, starting at 0 mm with 5 mm length intervals. Each foot was classified into the length class that was nearest to the foot length.

Foot scans were captured with 712 foot scanners; therefore, it was impossible to ensure all sales associates gave proper instructions to customers before scanning their feet. Some scanners were not attended by sales associates at all times; therefore, customers could perform scans on their own, without receiving any instructions. For these reasons, the foot scan data set had to be cleaned in order to remove problematic foot scans. Normal probability quantile-quantile (Q-Q) plots were used to identify the problematic foot scans. Images of 288 Q-Q plots were examined, one for each group, length class, and foot dimension. For each Q-Q plot, scans of foot measurements that substantially departed from a normal distribution were visually examined and problematic scans were removed from the data set. Most common reasons for removing scans were: scans of objects that were not human feet (human hands, dog legs, and others), pant legs interfering with foot measurements, scans with shoes, and scans of feet that had substantial abnormalities (missing toes, extreme hallux valgus, or extremely obese feet).

The following python libraries were used in this study: Pandas and Numpy for data manipulation and statistical data analysis, Statsmodels for plotting the Q-Q plots, and Seaborn for visualization of the results. Foot length kernel density estimates (KDE) of each region were plotted separately for male and female customers in order to compare foot length distributions between the studied regions. Line plots of mean values were plotted for each foot dimension separately for male and female customers in order to demonstrate the differences between the studied regions. Box-and-whisker plots were plotted for each foot dimension separately for male and female customers in order to demonstrate the dispersion of foot measurements. Line plots of mean values were plotted for each foot dimension separately for each region in order to demonstrate the differences between male and female dimensions.

## Results

### Number of foot scans

Feet classified to length classes 220 mm to 300 mm for male customers and 210 mm to 280 mm for female customers were included in this study. After removing feet that were out of the studied size ranges and removing the problematic scans from the foot scan data set, 1,200,847 3D foot scans were further analyzed. Numbers of scans per region and sex are presented in Table 2. The cumulative numbers of all scans per sex are displayed in the right column. The cumulative numbers of scans per region are presented in the bottom row. The large majority of customers were scanned in North America; however, the number of scans from the other two regions is still substantially higher than in any of the previous studies.

**Table 2:** Number of 3D foot scans per region and sex in the cleaned dataset.

	North America	Europe	Asia	Σ *regions*
male	494,833	43,064	10,072	547,969
female	610,675	31,470	10,733	652,878
all	1,105,508	74,534	20,805	1,200,847

### Foot length distribution

**Figure 2:**
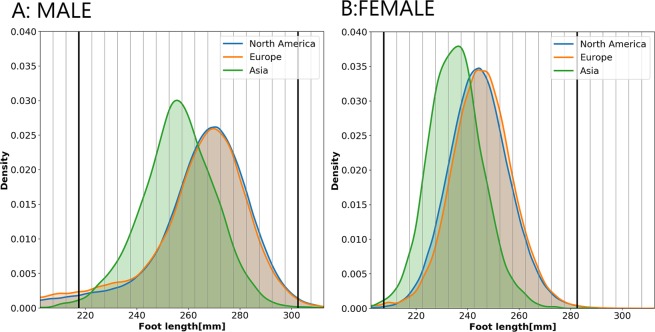
Kernel density estimate of foot length distribution. (**A**) Male foot length. (**B**) Female foot length.

Foot length distributions *i.e*. their kernel density estimates (KDE), for each region are presented in Fig. 2, separately for male (A) and female (B) customers. The scale is equal for both KDEs to enable comparison between male and female scans. The length distributions of North American and European feet are very similar, while those of Asian feet are shifted to the left, which means that Asian customers have substantially shorter feet than North American and European customers. The bold vertical lines in Fig. 2 indicate the lower and upper limits of foot length for the feet included in this study. The most frequently occurring length class for male customers was 270 mm for scans in North America and Europe, and 255 mm in Asia. For female customers, it was 245 mm for scans in North America and Europe, and 235 mm in Asia.

All further analyses were performed separately for each length class because of the proportional changes of foot measures according to size.

### Mean foot dimensions

**Figure 3:**
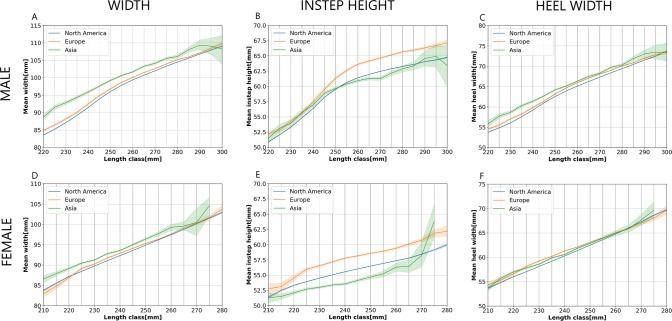
Mean foot dimensions (in mm) with 95% confidence intervals. (**A**) Mean foot width - male. (**B**) Mean instep height - male. (**C**) Mean heel width - male. (**D**) Mean foot width - female. (**E**) Mean instep height - female. (**F**) Mean heel width - female.

Figure 3 shows the mean foot dimensions (in mm) for male (A, B, C) and female (D, E, F) customers. The line plots show the mean values of the respective foot dimensions for every length class (solid lines) and the 95% confidence intervals around the estimated means (shaded areas around the solid lines). The depicted solid lines and the bounds of the confidence intervals (shaded areas) were interpolated for better visualization. Where two confidence interval shaded areas of two geographical regions are not overlapping for a given length class, the mean foot dimensions within that length class for the two regions are significantly different (at the level of significance p < 0.05). The same region color coding was used to label the regions in all line plots.

Figure 3(A) shows the mean foot widths per length class for male customers. Mean foot widths of the North American and the European male customers are significantly different in all length classes, even if the differences are very small (cca 1 mm). Male customers in Asia have noticeably and significantly higher mean foot width than customers in North America and Europe in all length classes (except 295 mm and 300 mm). The confidence intervals of the means for male Asian customers in the length classes 295 mm and 300 mm are quite wide for all foot dimensions, due to the low number of scans. Mean instep heights per length class for male customers are displayed in Fig. 3(B). Mean instep heights of North American male customers are significantly lower than mean instep heights of European male customers in all length classes. The Asian male customers have significantly different instep height than the North American customers in the length classes 225 mm, 230 mm to 245 mm, and 255 mm to 275 mm, but the differences are small. The Asian male customers have significantly lower instep height than the European customers in the length classes 245 mm to 290 mm, where the difference is up to 4 mm. Figure 3(C) shows mean heel widths per length class for male customers. Mean heel widths of North American male customers are significantly different than mean heel widths of European male customers in all length classes except in 300 mm, but the differences are very small. Asian male customers have significantly different heel widths than North American and European customers in the length classes 220 mm to 275 mm and 285 mm, with differences of up to 3 mm.

Mean foot widths of female customers in Asia are significantly higher than in North America and Europe in the length classes 210 mm to 260 mm (Fig. 3(D)). European female customers have significantly wider feet than North American female customers in the length classes 225 mm to 250 mm, but the differences are barely visible. There were no Asian female foot scans in the length class 280 mm. Figure 3(E) shows the mean foot instep heights for female customers. Similar to male customers, European female customers have significantly higher foot insteps than North American and Asian customers in a majority of the length classes (220 mm to 265 mm) and Asian female customers have lower instep heights than other customers in the length classes 210 mm to 265 mm. The differences in mean instep heights between European and Asian customers are especially large and go up to 4 mm. There are some significant differences in mean heel widths of female customers, but their magnitudes are quite negligible.

Mean foot widths, instep heights, and heel widths were foot length dependent. All mean foot dimensions in Fig. 3 have positive slopes; therefore, they are increasing with foot length for all regions and length classes. The exceptions are for the mean foot width and instep height of Asian male customers in the length classes 295 mm and 300 mm, where the 95% confidence interval was very wide due to a low number of customers. The slopes of the mean instep height for male customers in North America and Europe change substantially between the length classes 250 mm and 260 mm. The linear regression slope of the mean instep height of North American customers is 0.29 for the length classes 220 mm to 255 mm and 0.09 for the length classes 255 mm to 300 mm. The change of the slopes of the mean foot width for the same customers and length classes is smaller, but still noticeable; namely, from 0.42 to 0.25. The slopes of the mean instep heights are considerably lower than the slopes of the mean widths or the heel widths for both sexes and all regions. For instance, the linear regression slopes for mean instep height, foot width, and heel width of the North American feet in the length classes 255 mm to 300 mm are 0.09, 0.25, and 0.21. For North American female feet, the same slopes for the length classes 225 mm to 280 mm are 0.10, 0.26, and 0.23.

### Box plots of foot dimensions

**Figure 4:**
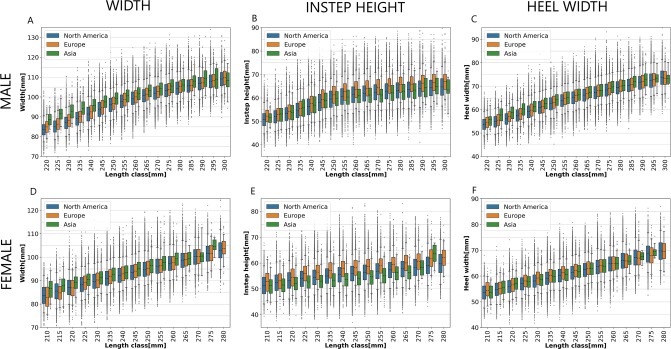
Box plots of foot dimensions. (**A**) Foot width box plot - male. (**B**) Instep height box plot - male. (**C**) Heel width box plot - male. (**D**) Foot width box plot - female. (**E**) Instep height box plot - female. (**F**) Heel width box plot - female.

In the process of foot measurement analysis, a large diversity of feet within the same region and length class was observed. Information about the diversity of foot measurements within one foot dimension would be very valuable for designing shoes and shoe lasts; therefore, further analysis was conducted. Box plots were used to graphically depict the dispersion of foot measurements within each foot dimension, length class, and region; the dispersion of measurements for male customers is presented in Fig. 4(A–C) and female customers in Fig. 4(D–F). The same color coding was used to label the regions as in Fig. 3. The lower side of a box is located at the first quartile (25^*th*^ percentile) and the upper side at the third quartile (75^*th*^ percentile) of all measurements; therefore, each box represents the middle 50% of the scanned feet within one length class and region. A horizontal line within the box of a box plot usually indicates the median of measurements; however, in Fig. 4, lines within the boxes indicate mean values in order to make the box plots comparable with the line plots in Fig. 3. Lines extending vertically from the boxes (whiskers) indicate the variability below the first quartile and above the third quartile. They extend to the 5^*th*^ percentile on the lower side and to the 95^*th*^ percentile on the upper side of the box. Ends of whiskers are indicated with short horizontal lines. A box together with both whiskers covers 90% of the scanned feet within one length class and region. Individual points below and above the whiskers represent outliers. Three box plots were plotted per length class, one for each region.

Foot width dispersion for male customers is depicted with box plots in Fig. 4(A). Differences between narrow and wide feet are substantial for all length classes and regions. For instance, the whiskers of North American feet in the 270 mm length class extend from 94.4 mm (narrow feet) to 110.2 mm (wide feet), while the mean foot width is 102.0 mm. The foot width span of 90% feet is 15.8 mm. Points below the bottom whisker in the same box plot represent 5% of the narrowest feet, and points above the top whisker represent 5% of the widest feet. The narrowest foot width is 84.1 mm and the widest 127.2 mm; the total foot width span of all feet is 43.1 mm.

Similar to the substantial foot width dispersion of male customers in Fig. 4(A) described above, considerable dispersion can be observed for all other foot dimensions: male instep height (B), male heel width (C), female foot width (D), female instep height (E), and female heel width (F).

**Figure 5:**
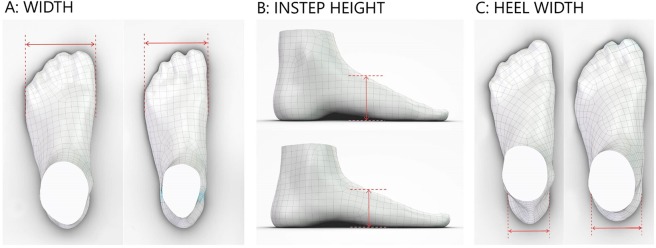
3D scans demonstrating dispersion of male North American feet in the 270 mm length class. (**A**) A narrow foot (5^*th*^ percentile) and a wide foot (95^*th*^ percentile). (**B**) A low instep foot (5^*th*^ percentile) and a high instep foot (95^*th*^ percentile). (**C**) A narrow heel foot (5^*th*^ percentile) and a wide heel foot (95^*th*^ percentile).

Figure 5 shows the dispersion of male North American feet in the 270 length class, namely a comparison of a narrow foot (5^*th*^ percentile, 94.4 mm) and a wide foot (95^*th*^ percentile, 110.2 mm) (A), a comparison of a low instep foot (5^*th*^ percentile, 54.6 mm) and a high instep foot (95^*th*^ percentile, 70.8 mm) (B), and a comparison of a narrow heel foot (5^*th*^ percentile, 61.2 mm) and a wide heel foot (95^*th*^ percentile, 73.9 mm) (C).

### Differences between male and female feet

**Figure 6:**
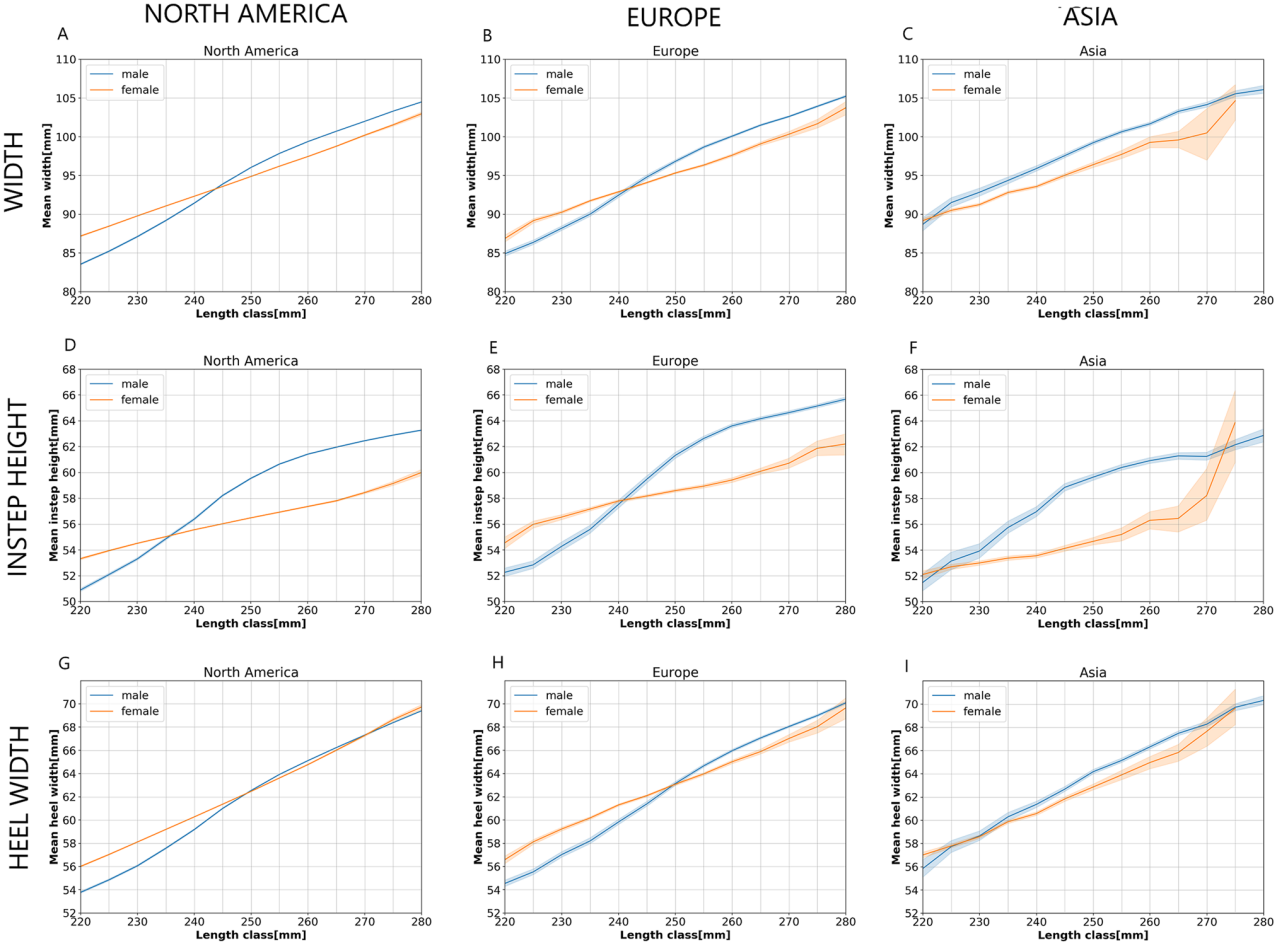
Differences between male and female feet. (**A**) Mean foot widths of North American customers. (**B**) Mean foot widths of European customers. (**C**) Mean foot widths of Asian customers. (**D**) Mean instep heights of North American customers. (**E**) Mean instep heights of European customers. (**F**) Mean instep heights of Asian customers. (**G**) Mean heel widths of North American customers. (**H**) Mean heel widths of European customers. (**I**) Mean heel widths of Asian customers.

Significant differences have been observed between the regions; therefore, the differences between male and female feet are analyzed separately for each region. Line plots of mean foot dimensions of male and female customers are plotted for each foot dimension and region, as depicted in Fig. 6. Shaded areas around the solid lines represent 95% confidence intervals. Length classes that overlap between the male and the female data set were used in this analysis, namely the length classes 220 mm to 280 mm.

Figure 6(A) shows a comparison between mean foot widths of the North American male and female customers per length class. Mean foot widths of male customers are significantly lower in length classes 220 mm to 240 mm and significantly higher than mean foot widths of female customers in the length classes 245 mm to 280 mm. Very similar conclusions can be drawn from Fig. 6(B) for the male and female European feet. The only change is that the foot width difference is not significant in the 240 mm length class. Mean foot widths of the male Asian customers are significantly higher than those of the female Asian customers for the length classes 230 mm to 265 mm, as depicted in 6(C). Figure 6(D) shows mean instep heights of the North American customers. Mean instep heights of the male customers are significantly lower in the length classes 220 mm to 230 mm and significantly higher in the length classes 240 mm to 280 mm. Mean instep heights of the European male feet are significantly lower in the length classes 220 mm to 235 mm and significantly higher than those of female customers in the length classes 245 to 280 (Fig. 6(E)). Mean instep heights of Asian male customers are significantly higher in the length classes 230 mm to 270 mm (Fig. 6(F)). Differences of mean heel widths between male and female feet are depicted in Fig. 6(G–I). Even though some differences are statistically significant, they are practically negligible.

## Discussion

The aim of this study was to compare the dimensions of human feet between the sexes and between different geographical regions, based on a large data set of 3D foot scans collected in a retail environment. Compared to previous similar studies, the number of subjects used in this study was larger by several orders of magnitude; however, scanning feet in a retail environment has its downsides as well. The lack of additional subjects’ attributes is the main drawback of this study compared to previous research. Age, height, weight, ethnicity, regular sport activities, foot injuries, foot conditions, usual footwear size, and other attributes would enable additional valuable analyses. For example, the availability of customers’ age would provide an opportunity to study how foot measurements change with age. Even though Q-Q plots were used to remove problematic feet from the dataset, it was not possible to visually examine all of the foot scans; therefore, some feet with foot conditions or injuries were probably still used in this study. However, collecting additional customer attributes in a retail environment would have a strong negative impact on the user experience; therefore, customers were not asked to provide this data during the scanning process. Foot lengths of scans that were captured on the same scanner within a time window of 3 minutes were analyzed in order to estimate the likelihood of scanning the same customer several times in a row. In less than 3% of scans, it was possible that the same customer was scanned more than once. However, the foot length distribution of the possibly repeated scans was very similar to the foot length distribution of the whole dataset; therefore, these scans didn’t substantially influence the results of this study.

The foot length distributions show substantial differences across regions and between the sexes. Customers in Asia have shorter feet than customers in North America and Europe for both sexes. Female customers have shorter feet than male customers in all regions. Foot length distributions may assist footwear retailers in stocking products in sizes and quantities that match their customers’ feet.

Long tails can be observed on the left side of the estimated male foot length distributions. While the reason for the tails is not completely clear, one explanation could be that they represent scans of boys’ feet, which is an important customer segment of one of the brands that is using the Volumental foot scanning solution in retail. Data from boys’ feet is also the most probable reason behind North American and European male feet appearing narrower than the female feet in the length classes 220 mm to 240 mm and having lower instep heights in the length classes 220 mm to 235 mm. It is very likely that the majority of male feet in these length classes are still growing and haven’t reached an adult shape. Boys’ feet couldn’t be excluded from the data set since the age of customers was not recorded.

Substantial foot differences between geographical regions have been reported by previous studies^9–11^. This study confirms that male and female Asian customers have significantly wider feet than European and North American customers, who have very similar mean widths. On the other hand, European customers have significantly higher instep height than North American customers, which hasn’t been reported in any of the previous studies. While the reason for this is not clear, one possible explanation could be that the lower mean instep height of American customers is caused by customers of Asian origin, who have lower insteps. Furthermore, lower insteps could be caused by American customers of other origins, like African and Latino American. Foot scans from regions with predominant African and Latino ethnic groups would be needed to confirm this conjecture.

Previous studies^3–8^ have reported substantial differences between male and female feet. This study confirms significant differences of mean widths, instep heights, and heel widths between male and female feet in all three geographical regions. The largest differences were observed for mean instep heights and widths, while the differences in heel widths were lower. The practical implication of the significant differences in foot dimensions among the 3 global regions and between male and female feet within each region is that shoes would have to be developed separately for each region and sex in order to take these factors into consideration.

The results of this study have demonstrated large dispersion of all studied foot dimensions in all regions. As depicted in Figs. 4(A) and 5(A), for the North American male feet in the 270 mm length class, the foot width span of 90% of feet is 15.8 mm. A shoe width increment in the US sizing system is 4.76 mm; therefore, more than three shoe widths are required to provide appropriate shoe width to 90% of the customers in this group. One shoe width can cover a maximum of 40.1% of these customers. Even though some brands are offering multiple widths of the same shoe style, the majority of shoes are available only in a single width. Instep height dispersion of the same group of customers is depicted in Figs. 4(B) and 5(B); the instep height span of 90% of feet is 16.2 mm. Most shoe styles have some kind of instep height adjustment system such as laces; however, shoe designers should more explicitly consider the high dispersion of instep heights. High dispersion has been demonstrated for heel width measurement; however, it is not clear how footwear brands are addressing it. Some outliers in Fig. 4 are far from the whiskers, which indicates that these customers are very unlikely to find well-fitting shoes off the shelf.

Considering the differences in mean values and the large dispersion of all studied foot dimensions, we can conclude that a shoe designed to fit the average feet of one geographical region and sex will not fit the average feet of another region or sex; however, it will fit some feet of the other region or sex. For example, a shoe designed to fit average male European feet will fit male customers in North America who have high instep. A shoe designed for average male feet in the length classes 250 mm to 280 mm will fit female feet that are wide and have high instep.

Finally, the slopes of all mean measurements in Fig. 3 may assist footwear brands in improving the current grading tables, which are used for developing shoes and shoe lasts of all sizes from a sample size shoe and shoe last. Current grading tables only provide width or girth scaling factors; therefore, shoes and lasts are graded in both width and height with the same scaling factor. However, the differences between the instep height slopes and both widths slopes indicate that the height scaling factors should be much lower than the forefoot and the heel width scaling factors when grading shoes and shoe lasts. The slopes of the mean measurements in Fig. 3 depend on the length class. Changes of the slope are the largest in the mean instep height lines, and also noticeable in the mean width lines; therefore, height and width scaling factors should not be constant across the whole size range, as they most commonly are in current grading tables. These insights can help improve shoe and shoe last scaling factors when grading shoes and shoe lasts from the sample size to the whole size range for all length classes, in all regions, and for both sexes.

In summary, shoes should be developed separately for each region and sex in order to take into account the differences in foot measures between the regions and the sexes. Shoes for the Asian market should be made wider compared to the shoes for the North American and the European market, and shoes for the European market should be made higher in the instep compared to the shoes for the Asian and the North American market. Male shoes for all 3 regions should be made wider and higher in the instep compared to female shoes. For all regions and both sexes, shoes should be produced in several widths in order to provide proper fit to a larger percentage of the customers. Finally, grading tables should take into account the findings of this study; scaling factors should not be linear across the whole size range, and height scaling factors should be lower than width scaling factors.

## Data Availability

The raw foot scan data used in this study are not publicly available, because the data are owned by third parties. The processed data are, however, available from the corresponding author upon reasonable request.
